# Impact of Gastroesophageal Reflux Disease (GERD) Symptoms on the Lifestyle and Academic Performance of Medical Students at King Faisal University

**DOI:** 10.7759/cureus.51261

**Published:** 2023-12-29

**Authors:** Omar Alomair, Ajlan Alajlani, Miad Abdullah M Abu Mughaedh, Majed M Almajed, Ahmed K Abu sinah, Sayed Ibrahim Ali

**Affiliations:** 1 College of Medicine, King Faisal University, Al-Ahsa, SAU; 2 Department of Medicine, King Khalid University, Abha, SAU; 3 College of Medicine, King Faisal University, Dammam, SAU; 4 Family and Community Medicine, King Faisal University, Hofuf, SAU

**Keywords:** lifestyle, king faisal university, medical students, academic performance, gastroesophageal reflux disease

## Abstract

Background

A number of symptoms and complications are associated with gastroesophageal reflux disease (GERD), which originates when stomach contents are refluxed into the esophagus. GERD has been associated with quality of life (QoL) issues as well as health-related problems. However, the evidence of this correlation among medical students is still unproven. This study aims to assess GERD severity and symptoms among medical students and investigate the effects of GERD on academic performance and quality of life among Saudi Arabian medical students.

Methods

This is a cross-sectional study design based on questionnaires distributed among medical students at King Faisal University, Saudi Arabia: Reflux-Qual Short form (RQS) and Frequency Scale for the Symptoms of GERD (FSSG). Demographic data include gender, academic year, and Grade Point Average (GPA). The participants included 382 studentsrandomly selected as the sample size, with a precision of 5% and a 95% confidence interval (CI).

Results

Data were collected from 382 medical students of King Faisal University. A total of 382 participants (215 (56.3%) females and 167 (43.7%) males) were evaluated. Among 382 students,325 (85.1%) were negative for GERD, while 57 (14.9%) had symptoms of GERD*. *In this study, the most frequent symptoms were feeling full while eating meals (8.9%), feeling of heaviness after meals (6.5%), bloating of the stomach (3.7%), and burping (3.7%). The most affected life domains were life satisfaction (18.1%), enjoyment of food (8.4%), avoidance of large meals (6.3%), and worrying about digestive problems (5.8%). This study shows a significant relationship between GERD and GPA (P < 0.005) and a significant negative relationship between GERD and QoL (P < 0.001).

Conclusions

GERD significantly affects the quality of life for medical students, primarily those with high GPAs. More research is needed to determine the reason behind this.

## Introduction

Gastroesophageal reflux disease (GERD), a common chronic disease, is caused by the reflux of stomach contents into the esophagus, resulting in several upper gastrointestinal signs and symptoms, most commonly heartburn and acid regurgitation, which may affect the quality of life (QoL) [1]. GERD is also connected to several additional symptoms that start in the chest, respiratory system, and esophagus; atypical symptoms, such as a persistent cough and hoarseness, chest discomfort, and wheezing, might afflict up to one-third of GERD patients. Even mild GERD has been shown to affect QoL [1,2], with effects such as reduced physical activity, disrupted sleep, and a decrease in overall well-being [1-3].

Impaired QoL can lead to lower work productivity, often resulting from sleep disruption brought on by nocturnal symptoms. It is well-recognized that insomnia causes daytime weariness and poor performance and has been linked to decreased productivity at work. People with nighttime GERD frequently experience sleep disruption, which may have a significant impact on the QoL [3-5]. Due to their esophageal and extra-esophageal symptoms, many patients with GERD regularly struggle to go about their everyday lives due to pain, fatigue, and feelings of poor physical and mental health. As a result, GERD may lead to both direct medical costs and indirect costs from reduced work productivity [5,6].

According to the most recent research, GERD affects populations in Europe, the United States, and the Middle East at a rate of 10 to 30% [7]. Countrywide research in Saudi Arabia indicates a prevalence of 28.7% [8]. Some studies found that the students are at higher risk of GERD compared to other populations [9,10]. This could be explained by risk factors such as stress [11,12]. However, the evidence of this correlation among medical students is still unproven. The establishment of the relationship between GERD and its effects on QoL and academic performance among medical students could increase awareness and reduce the risk factors. Therefore, this study aims to assess the severity of GERD symptoms among medical students and establish the relationship between GERD and QoL and academic performance among medical students in Saudi Arabia.

## Materials and methods

Study design

This is a cross-sectional study conducted at the Faculty of Medicine, King Faisal University, surveying both male and female students, using the Frequency Scale for the Symptoms of GERD (FSSG) questionnaire and Reflux-Qual Short form questionnaire (RQS) conducted online through Google Forms. The survey was disseminated through social media apps, primarily WhatsApp. The duration was six months (from May to October 2022). Ethical approval was received from King Faisal University's Research Ethics Commission; the reference number of ethical approval is KFU-REC2022-APR-EA000606.

Sample size

The established equation was used to determine sampling size n = (z^2^ × p × q)/d^2^, where n = the minimum sample size, Z =1.96, p = 0.5, and q = (1 − p) =0.5 [10]. This equation indicated that 382 samples are needed to obtain a precision of ±5% with a 95% confidence interval (CI).

Inclusion and exclusion criteria

All medical students at King Faisal University were included. However, medical interns were excluded from the study.

Questionnaire

Gender, academic year, and Grade Point Average (GPA) were among the demographics collected in the survey. We used the Reflux-Qual short form (RQS), which consists of eight items, giving a score between 0 and 32, with higher scores indicating a higher quality of life. A 12-item Gastroesophageal Reflux Symptom Frequency Scale (FSSG) questionnaire was used to evaluate the presence of GERD. FSSG scores over 8 indicated the presence of GERD.

Statistical analysis

Statistical Package for Social Sciences (SPSS) version 26.0 (IBM Corp., Armonk, NY) from IBM was used to conduct the necessary statistical analyses once the data were collected, validated, and entered into the computer. Continuous variables were represented as mean ± standard deviation (SD). For categorical variables, frequency was utilized. Categorical variables were also compared using chi-square. Statistical significance was defined as a p-value less than or equal to 0.05.

## Results

Participant demographics are shown in Table 1. Of note, 97.6% of participants have a very good or excellent GPA. The number of participants was 382, with a mean age of 22.15 ± 3.24 SD.

**Table 1:** Demographic data of the study participants

		n	%
Gender	Male	167	43.7
	Female	215	56.3
Academic year	First year	204	53.4
	Second year	28	7.3
	Third year	65	17.0
	Fourth year	51	13.4
	Fifth year	20	5.2
	Last year	14	3.7
What is your current GPA?	Good or lower than (3.5)	9	2.4
	Very good (3.5-4.4)	123	32.2
	Excellent (4.5-5)	250	65.4

Table 2 shows the aggregate results of the FSSG survey. In this study, the most frequent symptoms experienced “always” by the survey participants were: feeling full while eating meals (8.9%), feeling of heavy stomach after a meal (6.5%), bloating of the stomach (3.7%), and burping (3.7%). The least frequent symptoms were: subconsciously rubbing chest with hand (1.0%), burning sensation in throat (1.3%), epigastric pain before meals (1.6%), acid regurgitation (1.8%), epigastric pain after meals (1.6%).

**Table 2:** Symptoms in the study participants

	Never	Occasionally	Always
Do you get heartburn?	266(69.6%)	108(28.3%)	8(2.1%)
Do you sometimes subconsciously rub your chest with your hand?	274(71.7%)	104(27.2%)	4(1%)
Do you get heartburn after meals?	264(69.1%)	109(28.5%)	9(2.4%)
Does something get stuck when you swallow?	284(74.3%)	90(23.6%)	8(2.1%)
Do you get bitter liquid (acid) coming up into your throat?	236(61.8%)	139(36.4%)	7(1.8%)
Do you get heartburn if you bend over?	287(75.1%)	87(22.8%)	8(2.1%)
Do you have unusual (e.g., burning) sensations in your throat?	282(73.8%)	95(24.9%)	5(1.3%)
Does your stomach get bloated?	252(66%)	116(30.4)	14(3.7%)
Does your stomach ever feel heavy after meals?	97(68.1%)	97(25.4%)	25(6.5%)
Do you ever feel sick after meals?	238(62.3%)	136(35.6%)	8(2.1%)
Do you feel full while eating meals?	242(63.4%)	106(27.7%)	34(8.9%)
Do you burp a lot?	232(60.7%)	136(35.6%)	14(3.7%)
Do you get epigastric pain (burning) after meals?	263(68.8%)	113(29.6%)	6(1.6%)
Do you get epigastric pain (burning) before meals?	295(77.2%)	80(20.9%)	7(1.8%)

Among the 382 participants in this study, a majority (325 or 85.1%) were negative for GERD, according to the FSSG questionnaire, while 57 (14.9%) had significant GERD symptoms (positive GERD) (Table 3).

**Table 3:** GERD categories GERD: Gastroesophageal Reflux Disease

		Frequency	Percent
GERD categories	Negative GERD	325	85.1
	Positive GERD	57	14.9
	Total	382	100.0

The participants’ responses about quality of life, as affected by digestive problems, are shown in Table 4. Note that these data refer to the total sample of 382 students, not those identified as GERD-positive.

**Table 4:** Domains affected in life

	Extremely	Quite a bit	Moderately	Slightly	Not at all
During the past 4 weeks, have you been bothered by your digestive problem when gardening, or doing odd?	13(3.4%)	33(8.6%)	53(13.9%)	110(28.8%)	173(45.3%)
During the past 4 weeks, have you been bothered by your digestive problem? Have you done less than usual?	12(3.1%)	26(6.8%)	88(23%)	86(22.5%)	170(44.5%)
During the past 4 weeks, have you felt satisfied with your life in general even though you have a digestive problem?	109(28.5%)	94(24.6%)	68(17.8%)	42(11%)	69(18.1%)
During the past 4 weeks, considering your digestive problem, have you enjoyed food?	134(35.1%)	112(29.3%)	68(17.8%)	36(9.4%)	32(8.4%)
During the past 4 weeks, have you been worried because of your digestive problem?	22(5.8%)	46(12%)	82(21.5%)	80(20.9%)	152(39.8%)
During the past 4 weeks, because of your digestive problems, have you been in a bad mood?	16(4.2%)	48(12.6%)	109(28.5%)	86(22.5%)	123(32.2%)
During the past 4 weeks, have your digestive problems kept you awake most of the night?	7(1.8%)	18(4.7%)	58(15.2%)	78(20.4%)	221(57.9%)
During the past 4 weeks, have you avoided eating large meals because you were afraid of having digestive problems?	24(6.3%)	42(11%)	86(22.5%)	67(17.5%)	163(42.7%)

Table 5 shows that there was no significant relationship between GPA and QoL (P=0.101).

**Table 5:** QoL domains QoL: Quality of Life

		What is your current GPA?
QoL	Pearson Correlation	0.084
	P-value	0.101
	N	382

Table 6 shows that there was no significant differences were found between GPA categories and QoL score (P=0.159).

**Table 6:** GPA categories and QoL score GPA: Grade Point Average; QoL: Quality of Life

GPA	N	Mean	Std. Deviation	p-value	
					Good or lower than (3.5)	9	22.78	4.494	0.159	
Very good (3.5-4.4)	123	21.76	6.214						
Excellent (4.5-5)	250	23.01	5.844						

There is a significant relationship between GERD and GPA (P=0.005) (Table 7).

**Table 7:** Relationship between GERD and GPA GERD: Gastroesophageal Reflux Disease; GPA: Grade Point Average

		GERD_categories		p-value
		Negative gerd	positive gerd	
What is your current GPA?	Good or lower than (3.5)	6	3	0.005
		66.7%	33.3%	
	Very good (3.5-4.4)	96	27	
		78.0%	22.0%	
	Excellent (4.5-5)	223	27	
		89.2%	10.8%	

There is a significant negative relationship between GERD and QoL (P=0.001) (Table 8).

**Table 8:** The relationship between GERD and QoL **. Correlation is significant at the 0.01 level (2-tailed)

		QoL
GERD	Pearson Correlation	-.177^**^
	p-value	0.001
	N	382

## Discussion

The study included a relatively diverse sample of 382 participants, with a majority of female participants and participants in their first year of academic study. The majority of participants reported having an excellent GPA.

Using the FSSG questionnaire, the study assessed symptoms related to GERD among participants. The results showed that the majority of participants reported never experiencing GERD symptoms, while a smaller proportion reported occasionally experiencing these symptoms. Only a small proportion of participants reported always experiencing GERD symptoms. These findings are consistent with previous studies that have reported a low prevalence of GERD symptoms in the general population, with some studies reporting rates as low as 10-20% [13]. It Is important to note, however, that the prevalence of GERD symptoms can vary depending on the population being studied and the methodology used to assess these symptoms. For example, some studies have reported higher rates of GERD symptoms in specific populations, such as individuals with obesity or those with a history of smoking or alcohol consumption [14,15]. Additionally, the use of self-reported symptoms to diagnose GERD may not be as accurate as other diagnostic methods, such as endoscopy or pH monitoring.

The majority of participants with GERD did not experience significant negative effects on their quality of life as a result of their digestive problems. Specifically, most participants did not report being bothered by their digestive problems during activities such as gardening or doing housework, and many did not report doing less than usual or feeling dissatisfied with their overall quality of life. These findings are generally consistent with previous research that has explored the impact of digestive problems on quality of life. For example, a study by L’Heureux-Bouron et al. (2018) found that while digestive problems can cause discomfort and inconvenience, they do not necessarily have a significant negative impact on overall quality of life [16]. This may result from the ability to manage many digestive issues with dietary changes, medication, or other lifestyle modifications. Additionally, the body has a remarkable ability to adapt to digestive discomfort, and most people can continue to perform their daily activities despite experiencing digestive symptoms. Similarly, a study by Fikree and Byrne (2021) found that while digestive problems can have an impact on physical functioning, they do not necessarily lead to significant impairments in emotional well-being or overall quality of life [17]. However, it is worth noting that some participants in our study reported experiencing negative effects on their quality of life as a result of their digestive problems, such as being worried or in a bad mood. This is consistent with previous research that has found that some individuals with digestive problems may experience more severe symptoms and greater impairments to their quality of life than others [18]. The severity of digestive symptoms and their impact on an individual’s quality of life can vary depending on factors such as underlying causes, duration and frequency of symptoms, genetics, lifestyle factors, and coexisting medical conditions. Some individuals may experience more severe symptoms and greater impairment of their quality of life than others due to these factors.

Based on the correlation analysis between GPA and QoL, our study found a weak positive correlation between these two variables, which is consistent with some previous studies [19,20]. However, other studies have reported a stronger positive correlation between GPA and QoL [21,22]. It is worth noting that the size of the correlation coefficient in our study is very small, which suggests that the relationship between GPA and QoL may not be significant at a practical level.

Regarding the relationship between GPA and GERD, our study found a statistically significant negative association between these two variables, which suggests that a higher GPA is associated with a lower prevalence of GERD. This finding is consistent with some previous studies [23,24], although the specific percentages of students with positive GERD in each GPA category may vary across different studies. It is worth noting that our study used a cross-sectional design, which means that it is not possible to establish causality between GPA and GERD. Lastly, our study found a weak negative correlation between GERD and QoL. As GERD becomes more severe, it can have a greater negative impact on an individual’s quality of life. This is because more severe symptoms can be more frequent, persistent, and disruptive to daily activities such as eating, sleeping, and working. In addition, more severe GERD may require more aggressive treatment, which may have side effects that impact quality of life.

This research study has several limitations that should be acknowledged. The sample composition was skewed towards females and first-year students, limiting the generalizability of the findings. The reliance on self-reported symptoms may introduce subjective biases and recall inaccuracies. The study did not extensively explore potential confounding factors that may influence GERD symptoms and their impact on quality of life. The cross-sectional design used in the study prevents establishing causal relationships between variables. Additionally, the study’s specific context may not fully capture the range of experiences across different populations or cultural contexts.

## Conclusions

In this study, GERD-related symptoms are evaluated for the first time at King Faisal University regarding their impact on lifestyle and academic performance. According to our data, 14.9% of subjects have GERD. A total of 89% of subjects who have an excellent GPA are GERD-free. Considering the GPA and lifestyle of the subjects in our study, the impact is relatively high. When assessing the effects of GERD on GPA, we found, among all students, lower academic performance in GERD-positive students. On the basis of real and objective data about GERD and its relationship to lifestyle and GPA, this study may be considered as a preliminary study to assess the impact of different factors on gastrointestinal diseases.
